# Identification of T helper (Th)1- and Th2-associated antigens of *Cryptococcus neoformans* in a murine model of pulmonary infection

**DOI:** 10.1038/s41598-018-21039-z

**Published:** 2018-02-08

**Authors:** Carolina Firacative, A. Elisabeth Gressler, Kristin Schubert, Bianca Schulze, Uwe Müller, Frank Brombacher, Martin von Bergen, Gottfried Alber

**Affiliations:** 1 Institute of Immunology/Molecular Pathogenesis, Centre for Biotechnology and Biomedicine, College of Veterinary Medicine, University of Leipzig, Leipzig, Germany; 2 Group of Microbiology, National Institute of Health, Bogota, Colombia; 3 Department of Molecular Systems Biology, Helmholtz-Centre for Environmental Research, Leipzig, Germany; 4 International Centre for Genetic Engineering & Biotechnology (ICGEB), Cape Town component, South Africa and University of Cape Town, Division Immunology, Institute of Infectious Diseases and Molecular Medicine (IDM) & South African Medical Research Council (SAMRC), Cape Town, South Africa; 5 Institute of Biochemistry, Faculty of Biosciences, Pharmacy and Psychology, University of Leipzig, Leipzig, Germany; 6 Present Address: School of Medicine and Health Sciences, Universidad del Rosario, Bogota, Colombia; 7 Present Address: Research Group Microbial Immunology, Leibniz Institute for Natural Product Research and Infection Biology, Hans-Knöll-Institute (HKI), Jena, Germany

## Abstract

Cryptococcosis, caused by *Cryptococcus neoformans*, has been demonstrated to be controlled by T helper (Th)1 cells while Th2 cells are associated with fungal growth and dissemination. Although cryptococcal immunoreactive protein antigens were previously identified, their association with Th1 or Th2 immune responses was not provided. In mice, Th1-dependent IFN-γ induces the production of IgG2a, whereas the Th2 cytokine IL-4 stimulates the expression of IgG1 rendering each isotype an indicator of the underlying Th cell response. Therefore, we performed an immunoproteomic study that distinguishes Th1- and Th2-associated antigens by their reactivity with Th1-dependent IgG2a or Th2-dependent IgG1 antibodies in sera from *C. neoformans*-infected wild-type mice. We additionally analysed sera from Th2-prone IL-12-deficient and Th1-prone IL-4Rα-deficient mice extending the results found in wild-type mice. In total, ten, four, and three protein antigens associated with IgG1, IgG2a, or both isotypes, respectively, were identified. Th2-associated antigens represent promising candidates for development of immunotherapy regimens, whereas Th1-associated antigens may serve as candidates for vaccine development. In conclusion, this study points to intrinsic immunomodulatory effects of fungal antigens on the process of Th cell differentiation based on the identification of cryptococcal protein antigens specifically associated with Th1 or Th2 responses throughout mice of different genotypes.

## Introduction

*Cryptococcus neoformans*, an encapsulated basidiomycetous yeast, is the main etiological agent of cryptococcosis, a systemic and potentially fatal fungal infection. *C. neoformans* is ubiquitously present in the environment, especially in pigeon guano, which is the main known ecological niche of this pathogen^1,2^. Pulmonary infection with *C. neoformans* usually occurs through inhalation of infectious spores or desiccated yeasts from the environment establishing a normally latent, asymptomatic or minimally symptomatic disease in immunocompetent individuals^1–3^, although the risk to develop chronic allergic diseases such as asthma, has been shown to be enhanced in rats and BALB/c mice experimentally infected with *C. neoformans*^4,5^. In contrast, in immunocompromised persons such as AIDS patients, solid organ transplant recipients, or patients receiving exogenous immunosuppression, unresolved or untreated pulmonary cryptococcosis may lead to dissemination affecting the central nervous system (CNS) and causing meningitis or meningoencephalitis with a high mortality rate^1,6^. With about a quarter of a million individuals affected with cryptococcal meningitis per year and over 180,000 attributable annual deaths, this fungal infection is still responsible for 15% of all AIDS-associated mortalities^7^.

It is well known that the main host defence mechanism to resolve cryptococcosis is cell-mediated immunity by suppressing the growth of the yeasts in the lungs, which impedes dissemination to the CNS^8^. While T helper (Th)1 cells play a central role in induction of a protective immune response against cryptococcal infection, Th2 cells producing interleukin (IL)-4, IL-13, and IL-5, are detrimental in infection with *C. neoformans*^9,10^. Interestingly, *C. neoformans* is able to subvert immunoprotection by suppressing cellular immune response and through induction of humoral Th2 cell mediated immunity, resulting in a permissive environment for cryptococcal growth, characterized by IL-4-dependent immunoglobulin (Ig)E production, IL-13 dependent mucus production by goblet cells, IL-5-dependent eosinophilia, and functional pulmonary impairment, which are also features typically described in asthma^2,4,5,11^. Despite the benefits of available antifungal drugs, the emergence of drug-resistant fungal strains and several side-effects resulting from long term medication and toxicity, limit their use^12^. Therefore, adjunctive immunotherapy together with antifungal treatment is a promising option for the future^11^. The identification of cryptococcal protein antigens is of great interest for the development of an antifungal vaccine^11^. Particularly the discrimination between antigens that induce protective cell-mediated immunity responses to cryptococcal infection and antigenic compounds that are detrimental in cryptococcosis could contribute to the identification of vaccine candidates and targets for specific immunotherapy, respectively, which may help to reduce fungal burden, preventing the spread of the yeast from the lungs and increase the survival rate of the patients^11^.

Previous studies have aimed to identify protective cryptococcal proteins and protein fractions by their reactivity with antibodies produced by mice immunized with a murine IFN-γ-expressing *C. neoformans* strain (H99γ) that were in consequence protected against a subsequent challenge infection with a *C. neoformans* wild-type strain^13,14^. In another study, immunoreactive proteins of *C. gattii*, the closest related species to *C. neoformans* and the second most common etiological agent of cryptococcosis, have been identified using antibodies from sera of naturally infected koalas^15^. However, those studies lack the specific discrimination between immunoprotective and immunopathologic properties of the antigen that are associated  with either a Th1 or a Th2 response. We decided to use a proteomic approach involving two-dimensional (2D) gel electrophoresis and subsequent immunoblot, for the identification of Th1- and Th2-associated cryptococcal antigens based on the linkage of class switching in B cells with production of distinct Th cell cytokines^16^. Several murine studies firmly established that IL-4 regulates B cells for secretion of IgG1 antibodies, whereas interferon-γ stimulates the expression of IgG2a antibodies rendering either isotype an indicator of the underlying Th2 or Th1 response in mice^16–18^. Therefore, we chose to identify Th1- and Th2- associated antigens of *C. neoformans* by their reactivity specifically with either Th1-dependent IgG2a or Th2-dependent IgG1 from sera of infected wild-type and gene-deficient mice that lack either Th1 or Th2 responses. The proteomic approach allows separating the cellular proteins and the identification of immunoglobulins binding specific antigens^19^. Using this technique, distinct Th2- and Th1-associated fungal proteins were identified which are likely to play a role in shaping the Th cell response to *C. neoformans* and may be used for development of anti-fungal immunotherapy or vaccination regimens.

## Results

### Pulmonary infection with *C. neoformans* leads to dominant production of Th2-dependent IgG1 and IgE

**Figure 1:**
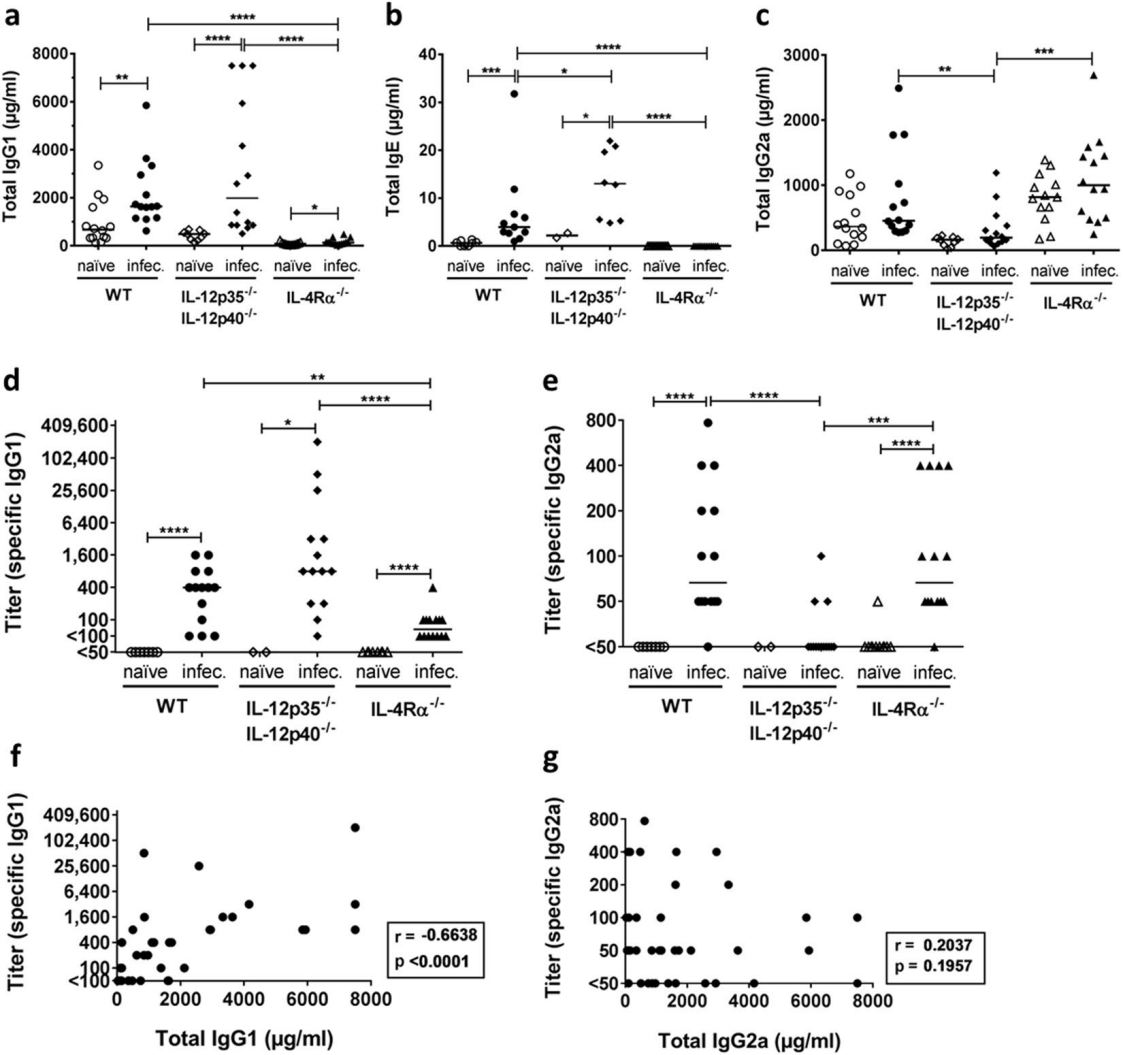
Total and *C. neoformans*-specific levels of Th2-dependent IgG1 and IgE predominate in sera from susceptible wild-type (WT) and IL-12-deficient mice with pulmonary cryptococcosis. Total IgG1 (**a**) and IgE (**b**) levels increased significantly after infection with *C. neoformans* for most genotypes, whereas IgG2a levels (**c**) were not influenced by *C. neoformans*. Compared to wild-type mice, IL-4Rα-deficient mice had markedly lower levels of immunoglobulin (Ig)G1 (**a**), no production of IgE (**b**) and similar levels of total IgG2a (**c**). Opposite, IL-12-deficient mice showed similar IgG1 and significantly higher IgE levels (**a**,**b**), while reduced levels of IgG2a (**c**), compared to wild-type mice. Titers of *C. neoformans*-specific IgG2a (**d**) and IgG1 (**e**) antibodies in infected (infec.) mice reflected the distribution observed for total immunoglobulin levels. *C. neoformans*-specific IgG1 and IgG2a antibodies were absent in sera of naïve mice. A strong correlation was observed between total and *C. neoformans*-specific IgG1 levels (**f**) but not between total and *C. neoformans*-specific IgG2a levels (**g**) in infected mice of all genotypes. Each spot represents a serum sample of an individual mouse (2 to 14 animals per group from at least two independent experiments) with the line indicating the median. Serum samples were obtained from mice intranasally infected with 500 colony forming units of *C. neoformans* strain 1841 after about 56 days post infection. Statistical significance determined by Mann-Whitney U test is shown as following: *p ≤ 0.05, **p ≤ 0.01, ***p ≤ 0.001, and ****p ≤ 0.0001. Correlation was determined by nonparametric Spearman’s correlation test.

In a BALB/c model of intranasal cryptococcal infection, IgG1/IgE and IgG2a have been shown to be valid indicators for preferential Th2 and Th1 responses, respectively^5,20^. Wild-type mice are susceptible to infection with the *C. neoformans* strain 1841^5,20^, while IL-4Rα-deficient mice, characterized by a dominant Th1 and Th17 response, are resistant to pulmonary cryptococcal infection^20^. In contrast, IL-12p35- and IL-12p40-deficient mice do succumb significantly earlier to intranasal infection (unpublished data) accompanied by a stronger Th2 response similarly as previously published for intravenously infected IL-12-deficient mice^21^. Sera from wild-type and knock-out mouse lines were used in order to rigorously distinguish specific Th1- and Th2-associated cryptococcal antigens. Levels of total IgG1 and IgE increased significantly in mice of all genotypes after infection with *C. neoformans*, except for IgE levels in IL-4Rα-deficient mice, which are unable to produce IgE^20^, underlining the overall Th2-biased immune response to this pathogen (Fig. 1a,b). In contrast, IgG2a levels were not influenced by cryptococcal infection (Fig. 1c). Comparison of total immunoglobulin levels revealed an increased Th2 response for IL-12p35- and IL-12p40-deficient mice, evidenced by the higher levels of total IgE in comparison with wild-type mice and comparable levels of IgG1 (Fig. 1a,b). Opposite, infected IL-4Rα-deficient mice showed a marked diminution in their Th2-associated B cell response, with no production of IgE and notably lower levels of total IgG1 compared to wild-type mice (Fig. 1a,b), consistent with previously published experiments^20^. Among all three groups of infected mice, production of total Th1-dependent IgG2a was determined to be lower in IL-12-deficient mice, while IL-4Rα-deficient mice produced the highest levels of this IgG isotype, although without statistical significance (Fig. 1c). When comparing the total levels of IgG1 and IgG2a in sera from infected IL-12- and IL-4Rα-deficient mice, a significant negative correlation between IgG1 and IgG2a was found (r = −0.5077, *p* = 0.0058**), confirming the opposite Th phenotypes in the selected mutant mice, while in sera from infected wild-type mice, there was no correlation between the two IgG isotypes (data not shown).

After quantification of total immunoglobulin concentrations, the titers of *C. neoformans-*specific IgG1 and IgG2a antibodies were determined, using a cryptococcal antigen-specific enzyme-linked immunosorbent assay (ELISA). Compared to wild-type and IL-12-deficient mice, titers for specific IgG1 antibodies against *C. neoformans* were lower in infected IL-4Rα-deficient mice (Fig. 1d), while infected IL-12-deficient mice showed reduced levels of specific IgG2a antibodies compared to infected wild-type and IL-4Rα-deficient mice (Fig. 1e). Overall, titers for *C. neoformans*-specific IgG1 antibodies reached higher values in comparison to *C. neoformans*-specific IgG2a titers. Furthermore, titers of *C. neoformans*-specific IgG1 and IgG2a showed a significant increase upon cryptococcal infection for all genotypes except for IgG2a detected in sera of IL-12-deficient mice (Fig. 1d,e). *C. neoformans*-specific IgG1 and IgG2a antibodies were absent in sera from naïve mice of all genotypes (Fig. 1d,e). A strong correlation was found between total and specific levels of IgG1 (r = −0.6638, ****p < 0.0001, Fig. 1f) but not between total and specific levels of IgG2a (r = 0.2037, *p* = 0.1957, Fig. 1g) in sera from infected mice of all genotypes, indicating an influence of *C. neoformans* on the production of IgG1 but not IgG2a antibodies. We did not determine *C. neoformans*-specific IgE titers in serum samples, as these are expected to be very low according to a previous study^22^. In conclusion, we could confirm the previously observed Th2-tilted immune response on the level of both, total and specific IgG1 and IgG2a antibodies upon *C. neoformans* infection.

### Proteomic analysis reveals cryptococcal antigens specifically reactive with IgG1 or IgG2a antibodies

After determining the titers for specific IgG1 and IgG2a antibodies against *C. neoformans* proteins, representative serum samples from each group of infected mice, five from wild-type and two from gene-deficient mice, were tested by immunoblot analyses following one-dimensional (1D) gel electrophoresis. Serum samples that showed high titers of *C. neoformans*-specific antibodies and titers around the median value were chosen. Additionally, serum samples from naïve mice (one per genotype) were included. As expected, detection of IgG1-reactive proteins after 1D sodium dodecyl sulfate polyacrylamide gel electrophoresis (SDS-PAGE) of cryptococcal proteins and subsequent immunoblotting revealed several bands following incubation with sera from infected wild-type mice (Supplementary Fig. S1). No bands were visible after incubation of the membrane with sera from naïve wild-type mice, naïve IL-12-deficient mice and both, infected and naïve IL-4Rα-deficient mice (Supplementary Fig. S1). IL-12p35-deficient mice produced strong IgG1 responses, indicated by an increased number of IgG1-reactive protein bands visible after incubation with sera (Supplementary Fig. S1).

Analysis of IgG2a-reactive proteins revealed one band after incubation of the membrane with sera from naïve mice of all genotypes, indicating nonspecific reactivity with cryptococcal proteins (Supplementary Fig. S1). As expected, incubation with sera from infected mice of all genotypes resulted in additional protein bands, demonstrating that IgG2a antibodies recognized several *C. neoformans* proteins.

**Figure 2:**
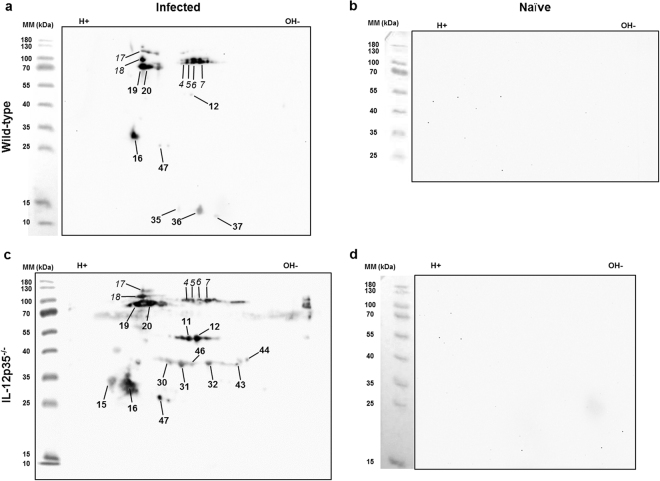
IgG1-immunoreactive proteins from *Cryptococcus neoformans* were detected with sera from representative infected but not naïve wild-type and IL-12-deficient mice. Whole cell proteins of *C. neoformans* strain 1841 separated by 2D electrophoresis were transferred to nitrocellulose membranes, which were thereafter incubated with sera from infected and naïve wild-type and gene-deficient mice diluted 1:1,000. IgG1-immunoreactive proteins were detected using sera from an infected wild-type (**a**), a naïve wild-type (**b**), an infected IL-12-deficient (**c**), and a naïve IL-12-deficient (**d**) mouse. Protein abundance as shown in the Coomassie staining did not correlate with the strength of the immunoreactive signal (Fig. 4). Only the spots that could be mapped on Coomassie-stained gels were numbered in the blot images. Bold numbers indicate strictly IgG1-reactive proteins while italic numbers mark proteins reactive with both, IgG1 and IgG2a antibodies. Images were cropped to improve clarity. Full-length blots without numbered protein spots are presented in Supplementary Fig. 2. Abbreviation: MM = molecular mass.

**Figure 3:**
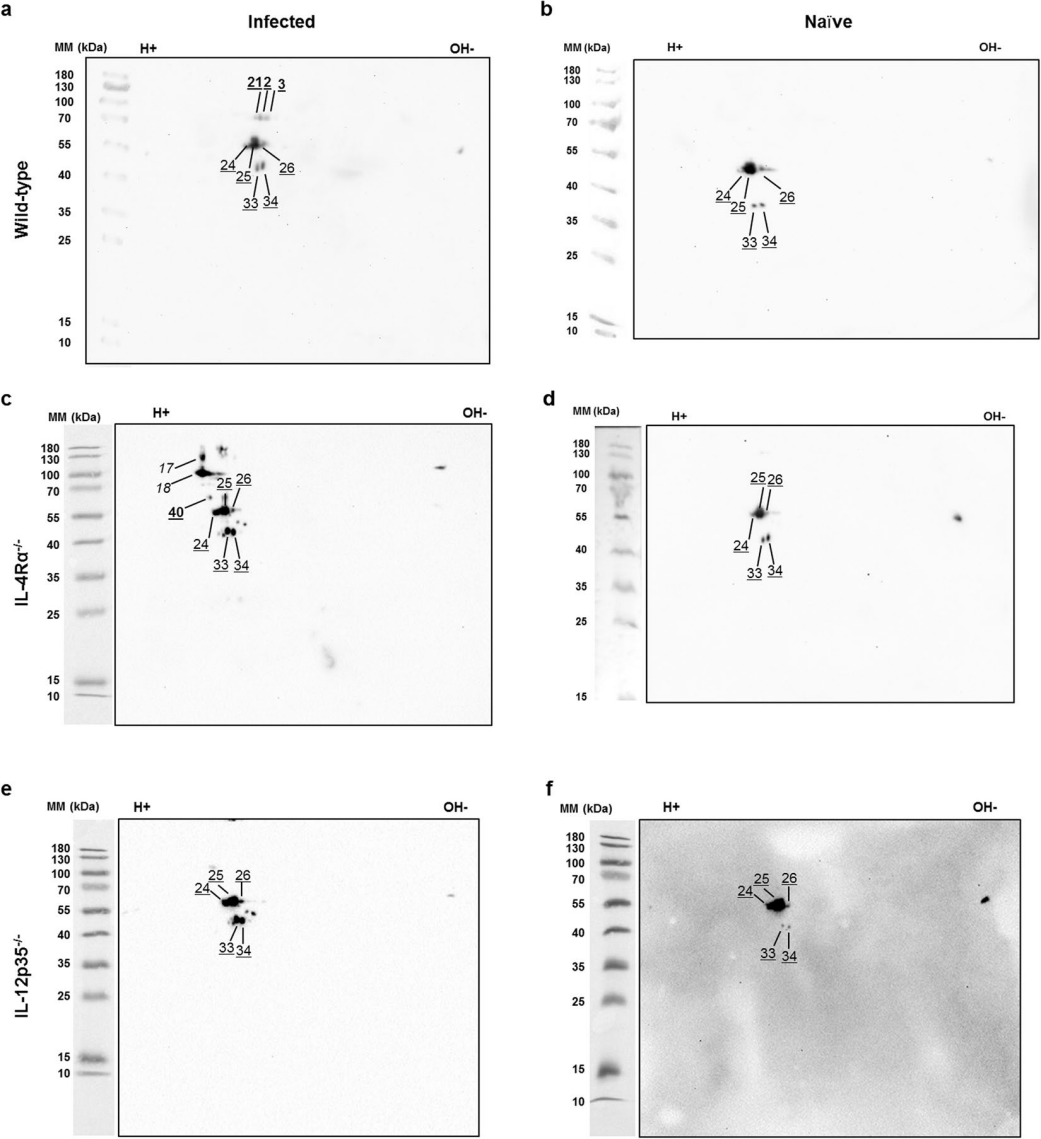
IgG2a-immunoreactive proteins from *Cryptococcus neoformans* were detected with sera from representative infected and naïve wild-type, IL-4Rα-deficient, and IL-12-deficient mice. Whole cell proteins of *C. neoformans* strain 1841 separated by 2D electrophoresis were transferred to nitrocellulose membranes, which were thereafter incubated with sera from infected and naïve wild-type and IL-4Rα-deficient mice diluted 1:1,000. IgG2a-immunoreactive proteins were detected using sera from an infected (**a**) and naïve (**b**) wild-type mouse, an infected (**c**) and naïve (**d**) IL-4Rα-deficient mouse, and an infected (**e**) and naïve (**f**) IL-12-deficient mouse. Protein abundance as shown in the Coomassie staining did not correlate with the strength of the immunoreactive signal (Fig. 4). Only the spots that could be mapped on Coomassie-stained gels were numbered in the blot images. Bold, underlined numbers mark IgG2a-reactive, *C. neoformans*-specific spots, while light, underlined numbers indicate IgG2a-reactive but *C. neoformans*-unspecific spots. Italic numbers mark spots reactive with both IgG1 and IgG2a antibodies. Images were cropped to improve clarity. Full-length blots without numbered protein spots are presented in Supplementary Fig. 3. Abbreviation: MM = molecular mass.

**Figure 4:**
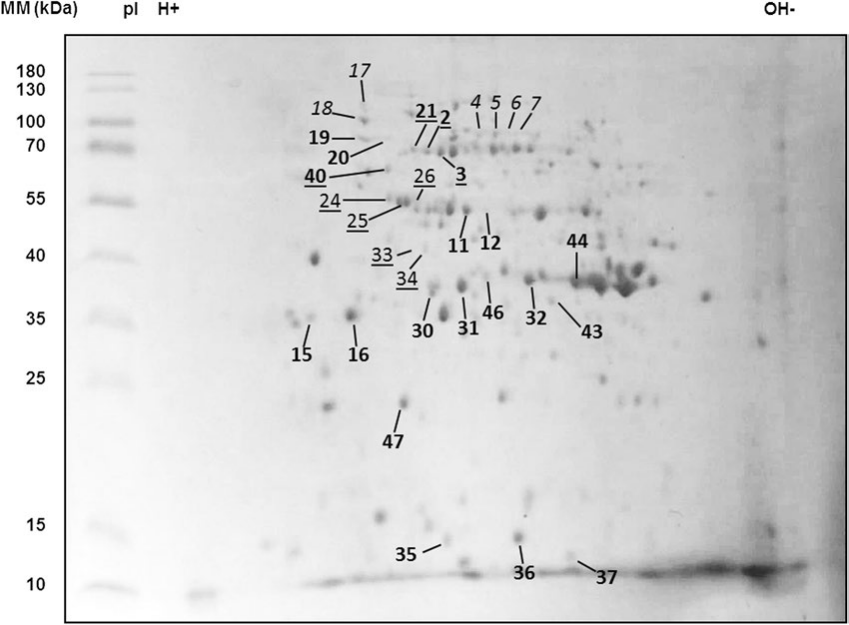
Protein profile of *Cryptococcus neoformans* with indicated immunoreactive protein spots. Whole cell proteins of *C. neoformans* strain 1841 were separated by isoelectric point and molecular weight. After 2D gel electrophoresis, gels were stained with Coomassie Brilliant Blue G250. Numbered spots in the stained gel represent all antigenic proteins that were identified in this study. Bold non-underlined and bold underlined numbers indicate IgG1- and IgG2a-immunoreactive proteins, respectively. The spots in italic were reactive with both isotypes as shown in Figures 2 and 3. Light underlined numbers indicate IgG2a-immunoreactive proteins, which were not specific for *C. neoformans*. Abbreviation: MM = molecular mass.

**Figure 5:**
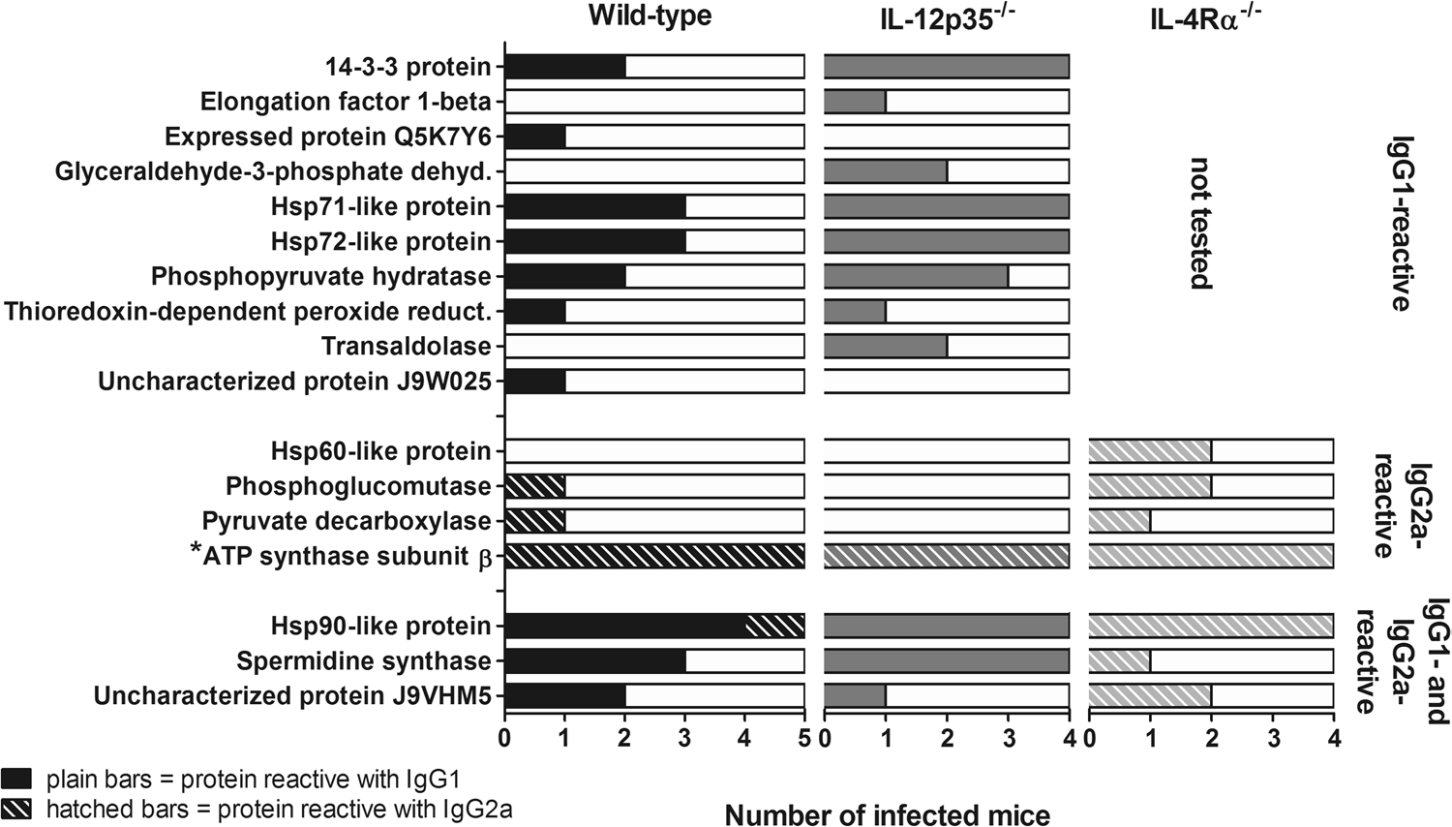
Frequency of cryptococcal proteins, reactive with IgG1, IgG2a, or with IgG1 and IgG2a antibodies in *Cryptococcus neoformans*-infected mice of different genotypes. Immunoreactive cryptococcal proteins were identified using sera from infected mice of different genotypes. Proteins were grouped according to their reactivity with IgG1, IgG2a, or with IgG1 and IgG2a antibodies. The isotype which showed reactivity in the individual animal is indicated by plain bars (IgG1) or hatched bars (IgG2a). Three IgG2a-reactive proteins reacted exclusively with sera from infected mice, while one protein also showed reactivity with sera from naïve mice (marked with an asterisk (*), see also Fig. 3). Sera from IL-4Rα-deficient mice were not tested by 2D analysis for IgG1-reactive proteins, as no immunoreactive protein bands for this isotype could be detected when investigating these sera in 1D analysis (Supplementary Fig. S1). Sera from infected animals were taken from at least three independent experiments in late infection state (at least 56 days post infection). Abbreviations: Glyceraldehyde-3-phosphate dehyd. = Glyceraldehyde-3-phosphate dehydrogenase; Thioredoxin-dependent peroxide reduct. = Thioredoxin-dependent peroxide reductase.

To achieve sufficient resolution for identification of individual *C. neoformans* proteins, 2D gel electrophoresis and subsequent immunoblot experiments were performed. We decided to analyse sera from five wild-type, four IL-12-deficient and four IL-4Rα-deficient mice. Only immunoreactive spots, which could be accurately mapped on the corresponding Coomassie-stained SDS gels were taken into account for further analysis. Representative immunoblots of individual mice, which display most but not all immunoreactive spots observed in a total of four to five mice, are shown in Figures 2 and 3. Nine IgG1-immunoreactive protein spots were detected using sera from infected wild-type mice (Fig. 2a, bold numbers). When using sera from infected IL-12p35-deficient mice, seven additional IgG1-immunoreactive protein spots were identified (Fig. 2c, bold numbers). Importantly, as already indicated by 1D gel analysis (Supplementary Fig. S1), no proteins were found to be IgG1-immunoreactive with sera from naïve wild-type and naïve IL-12p35-deficient mice (Fig. 2b,d). Most IgG1-immunoreactive spots could be identified in several individual mice of different genotypes (Fig. 5). Sera from IL-4Rα-deficient mice were not tested for IgG1-reactive antigens, as no immunoreactive bands were detected after 1D analysis (Supplementary Fig. S1). Nine protein spots were determined to be exclusively IgG2a-immunoreactive (Fig. 3a,c,e), from which five were considered IgG2a-immunoreactive but not *C. neoformans*-specific, as they also reacted with sera from naïve wild-type, IL-4Rα-deficient, and IL-12-deficient mice (Fig. 3b,d,f, light, underlined numbers). Three protein spots were identified as infection-specific when using serum from an infected wild-type mouse (Fig. 3a, bold, underlined numbers), which were not detectable using sera from IL-12deficient mice (Fig. 3e). In contrast, incubation with serum from infected IL-4Rα-deficient mice resulted in one additional spot (Fig. 3c). Notably, in contrast to IgG1-reactive proteins, infection-specific IgG2a-reactive spots only occurred in single animals without a high consistency (Fig. 5). To our surprise, only one protein spot (#18) was both IgG1- and IgG2a-immunoreactive in sera from infected wild-type mice. Few spots (#4–7, #17) were exclusively IgG1-immunoreactive in sera from infected wild-type mice (Fig. 2a,c, italic numbers), but showed reactivity with IgG2a when using serum from infected IL-4Rα-deficient mice (Fig. 3c, (spots #4–7 not shown)). In conclusion, this suggests that the host genotype in some cases (i.e. under strong Th cell polarizing conditions) affects the regulation of the Th-dependent isotype, but, in the wild-type host, fungal antigens significantly determine the Th cell phenotype. Immunoreactivity of the different proteins spots, seen by the intensity on the membrane, did not correlate with the abundancy of the proteins in the microorganism, as evidenced by Coomassie staining of the proteins in the gel (Fig. 4). This indicates that the given protein abundancy does not influence the degree of immunoreactivity.

**Table 1:** IgG1-immunoreactive proteins from *Cryptococcus neoformans*.

Protein (MW; UniProt ID)	Number of isoforms (spot #)	Immunological characteristics previously reported^a,b,c^	Ref.
14-3-3 protein, putative (29.0 kDa; Q5K8Z6)	1 (#16)	^a^Recognized as an antigen in mice infected with *C. neoformans* H99γ and in *C. gattii* infection in humans and koalas. ^c^Antibodies against this protein are induced in the course of the natural infection of schistosomiasis.	^13,15,26,52^
Elongation factor 1-beta (24.4 kDa; Q5KKD1)	1 (#15)	^a^Recognized as an antigen in patients with cryptococcosis caused by *C. gattii* and as a non-specific antigen in *C. gattii* infection in koalas.	^15,26^
Expressed protein Q5K7Y6 (14.9 kDa; Q5K7Y6)	1 (#35)	Not reported to date	
Glyceraldehyde-3-phosphate dehydrogenase (36,308 kDa; J9VRH1)	1 (#44)	^a^Recognized as an antigen in patients with cryptococcosis caused by *C. gattii*. ^b^ Immunoreactive protein identified in mice inoculated with *Candida albicans* and in sera from patients with paracoccidioidomycosis. ^b^This protein appeared not to be a suitable target for the development of immunotherapeutic strategies against candidiasis, despite being an immunodominant component that induces antibody response against *C. albicans*. ^b^The most abundant allergen from *Aspergillus fumigatus* in human sera. ^c^Common immunogenic antigen among *Eimeria* species. This protein was evaluated in form of DNA vaccine, which induced effective protection against single and mixed infection of these species. ^c^Antibodies against this protein are induced in infection of schistosomiasis. This protein is considered a target of protective immunity in humans against *Schistosoma mansoni* and *S. haematobium*.	^19,26,53–57^
Hsp71-like protein (69.6 kDa; J9VZ70)	1 (#19)	^a^70-kD Hsp family from *C. neoformans* described as a major target molecule of the humoral response in mice. ^a^Hsp70 recognized as an antigen in mice infected with *C. neoformans* H99γ and *C. gattii*. ^a^Hsp70 identified as immunodominant protein in mice immunized with *C. gattii* cell wall and cytoplasmic protein preparations.	^13–15,23,26,37,58^
Hsp72-like protein (69,513; J9VU43)	1 (#20)	^a^70-kD Hsp family from *C. neoformans* described as a major target molecule of the humoral immune response in mice.	^37^
Phosphopyruvate hydratase (Enolase) (47.7 kDa; Q5KLA7)	2 (#11, #12)	^a^Recognized as an antigen in mice infected with *C. neoformans* H99γ and in *C. gattii* infection in humans and koalas. ^a^Immunodominant protein identified in mice immunized with *C. gattii* cell wall and cytoplasmic protein preparations. ^b^Stimulates protective IgG2a in sera from vaccinated mice with systemic candidiasis. ^b^A major antigen of fungal infection (*A. fumigatus* and *C. albicans*)	^13–15,23,26,39,59^
Thioredoxin-dependent peroxide reductase (21.6 kDa; Q5KEB3)	1 (#47)	Not reported to date	^15^
Transaldolase (35.3 kDa; Q5K952)	4 (#30–32, #43, #46)	^a^Recognized as an antigen in mice infected with *C. neoformans* H99γ. ^a^Immunodominant protein identified in mice immunized with *C. gattii* cell wall protein preparations. ^b^Identified as an allergen of *Fusarium proliferatum* and *Cladosporium* and *Penicillium* species.	^13,23,60,61^
Uncharacterized protein J9W025 (14.8 kDa; J9W025)	2 (#36, #37)	Not reported to date.	

Ten cryptococcal immunogenic proteins were identified to react specifically with IgG1 antibodies from mice infected with *C. neoformans*.

MW: Molecular weight; UniProt ID: Identification number in the UniProt database.

^a^Described in proteomics studies of cryptococcosis, ^b^other mycoses, and ^c^other infections.

**Table 2:** IgG2a-immunoreactive proteins from *Cryptococcus neoformans*.

Protein (MW; UniProt ID)	Number of isoforms (spot #)	Immunological characteristics previously reported^a,b^	Ref.
***C. neoformans*** **-specific**			
Hsp60-like protein (61.4 kDa; J9VJ21)	1 (#40)	^b^Vaccination with recombinant Hsp60 protects mice against a lethal intravenous inoculum of *Histoplasma capsulatum* yeast cells. ^b^IgG1 and IgG2a monoclonal antibodies significantly prolonged the survival of mice infected with *H. capsulatum*.	^40,43^
Phosphoglucomutase (60.5 kDa; J9W313)	2 (#2, #21)	^b^Recognized as an antigen in *A. fumigatus*.	^39^
Pyruvate decarboxylase (67.6 kDa; J9VTH3)	1 (#3)	^b^Stimulates protective IgG2a in sera from vaccinated mice with systemic candidiasis. ^b^Recognized as an antigen of *Aspergillus fumigatus*.	^39,59^
***C. neoformans*** **-unspecific**			
ATP synthase subunit beta (58.7 kDa; J9VPP7)	5 (#24–26, #33–34)	^a^Recognized as an antigen in mice infected with *C. neoformans* H99γ, an antigen in humans infected with *C. gattii* and as a non-specific antigen in *C. gattii* infection in koalas.	^14,15,26^

Four cryptococcal immunogenic proteins were identified to react specifically with IgG2a antibodies. Three of them were determined as *C. neoformans*-specific and one was regarded as *C. neoformans*-unspecific, as it reacted strongly with sera from naïve wild type, IL-12- and IL-4Rα-deficient mice.

MW: Molecular weight; UniProt ID: Identification number in the UniProt database infec.: infected.

^a^Described in proteomics studies of cryptococcosis and ^b^other mycoses.

Mass spectra analysis of the 31 immunoreactive protein spots (Fig. 4) led to the identification of 17 unique cryptococcal proteins, as different isoforms of the same protein were identified in more than one spot. The frequency of these proteins in *C. neoformans-*infected mice of different genotypes is displayed in Fig. 5. Ten cryptococcal proteins were determined to be exclusively Th2-associated antigens as they reacted specifically with IgG1 antibodies (Table 1, Fig. 5), while only three proteins were exclusively immunoreactive with Th1-dependent IgG2a among the *C. neoformans*-specific proteins (Table 2, Fig. 5). From the five spots that were not *C. neoformans*-specific, but also exclusively IgG2a-reactive, one protein was identified (Table 2, Fig. 5). Identification of the six protein spots reactive with both, IgG1 and IgG2a antibodies (#4–7, #17, #18) revealed three immunoreactive proteins (Table 3, Fig. 5). Tables 1–3 give an overview of the immunological characteristics of these proteins or family of proteins as reported in previous studies of fungal infections including cryptococcosis. The proteins identified herein have various functions in cellular metabolism, growth as well as stress resistance and virulence. Eight out of the 17 identified proteins were previously identified in immunoproteomic studies on immunoreactive antigens of *C. neoformans* and *C. gattii*^13–15,23^. Taken together, using a proteomic approach we could identify ten IgG1- and three IgG2a-reactive *C. neoformans*-specific antigens using sera from infected wild-type and gene-deficient mice, which can be associated with either Th2- or Th1-mediated immune responses.

**Table 3:** *Cryptococcus neoformans* proteins immunoreactive with both, IgG1 and IgG2a antibodies.

Protein (MW; UniProt ID)	Number of isoforms (spot #)	Immunological characteristics previously reported^a,b^	Ref.
Hsp90-like protein (79.2 kDa; J9VVA4)	1 (#18)	^a^Hsp90 recognized as an antigen in mice infected with *C. neoformans* H99γ. ^b^A major antigen of *A. fumigatus*.	^13,14,39^
Spermidine synthase, putative (82.4 kDa; Q5KEA8)	4 (#4–7)	Not reported to date.	
Uncharacterized protein J9VHM5 (73.0 kDa; J9VHM5)	1 (#17)	Not reported to date.	

Three cryptococcal immunogenic proteins were identified to react with both IgG1 and IgG2a antibodies from mice infected with *C. neoformans*. Immunological characteristics of the proteins or protein family compared to previous studies of fungal infections, including cryptococcosis, are given.

MW: Molecular weight.

UniProt ID: Identification number in the UniProt database.

^a^Described in proteomics studies of cryptococcosis, and ^b^other mycoses.

## Discussion

Cryptococcosis remains one of the prominent infectious diseases in both, industrialized and developing countries. Even though overall outcome of antifungal therapy is effective, the rates of morbidity, mortality and relapse episodes among cryptococcosis patients continue to be remarkably high^7^. The search for alternative treatments for this mycosis and the prevention of cryptococcal dissemination by immunotherapy or vaccination is therefore of significant importance. Previously, attempts have been made to establish a protective vaccine against cryptococcosis by using capsular polysaccharides for immunization of mice, which turned out to elicit immunological unresponsiveness^24^. This could be overcome by linking cryptococcal polysaccharides to carrier proteins (i.e. using conjugate vaccines). In contrast to carbohydrate antigens, immunoreactive protein antigens are capable of eliciting direct T cell-dependent responses^25^, which is critical for the control of cryptococcal infection. In contrast to previous studies identifying immunoreactive cryptococcal antigens^13–15,26^, the immunoproteomic approach utilized in the present study is the first of its kind for the discrimination of cryptococcal Th2- and Th1-associated antigens seen by their reactivity with antibodies of the murine isotypes IgG1 or IgG2a, respectively. We confirmed the previously observed capacity of the fungus to induce a biased Th2 response in BALB/c mice^4,5^ demonstrated by the significantly increased levels of total IgG1 and IgE levels upon infection with *C. neoformans* and higher levels of IgG1 than IgG2a antibodies specific for cryptococcal antigens. We also identified a larger number of IgG1-reactive *C. neoformans*-specific antigens, which also occurred with enhanced consistency throughout different animals in comparison to IgG2a-reactive *C. neoformans*-specific antigens.

The induction of Th2-tilted immune responses by *C. neoformans* has been associated with cell wall and capsular components such as chitin and glucuronoxylomannan^27–29^. In addition to these carbohydrate factors, several proteins have been identified, such as (i) Pik1, Rub1 and Ena1, which deletion resulted in a decreased Th2-response upon infection^30^, (ii) laccase and urease, which promoted Th2 polarization^31,32^, or (iii) Ssa1, that was shown to promote macrophage M2 skewing during the afferent phase of the immune response against *C. neoformans*^33^. From these immunomodulatory proteins, the Hsp70 protein Ssa1 (annotated as Hsp71-like protein) was also identified in our study.

Interestingly, we were able to identify distinct Th1- and Th2-associated cryptococcal antigens throughout mice of different genotypes, which seemingly contrasts the immunological paradigm that the process of Th cell differentiation is mainly influenced by the surrounding cytokine milieu rather than the immunogenic antigen^34^. As the differentiation of T cells occurs after the interaction with antigen-presenting cells (APCs)^35^, distinct antigens may influence APCs to produce certain cytokines driving either Th1- or Th2-differentiation. It is conceivable that cellular vs. secreted cryptococcal proteins could target different APCs. However, presently it is not clear which of the identified proteins are either cell-associated or secreted. Other parameters influencing Th cell differentiation are the dose and binding strength of the antigen to the T-cell receptor resulting in different strength of T-cell receptor signalling and therefore distinct activation of downstream signals and transcription factors^34,36^. At this point it remains unclear how the identified Th1- and Th2-associated immunoreactive fungal antigens exert their influence on immune cells, but we hypothesize distinct direct interactions with APCs during the process of T cell differentiation. Future experiments therefore will include direct stimulation of APCs and T cells with the identified recombinantly expressed Th1- or Th2-associated *C. neoformans* antigens and furthermore the recombinant antigens will be used *in vivo* for immunization of mice.

IgG1-specific antigens are promising targets for specific immunotherapies addressed to restrain Th2-type responses, which are associated with exacerbation of disease, by skewing the Th cell differentiation towards a protective Th1 response. The IgG1-immunoreactive antigens identified in our study include proteins that are essential for growth and virulence of *C. neoformans* as they are involved in metabolism, oxidative stress, protein synthesis, and to maintain cell wall integrity (Table 1, Fig. 5). From those, six proteins, phosphopyruvate hydratase (enolase), elongation factor 1-β, 14-3-3 protein, Hsp71-like protein (Ssa1), transaldolase and glyceraldehyde-3-phosphate dehydrogenase, have been previously reported to be immunogenic in *C. neoformans* and its sibling species *C. gattii*^13–15,23,26^, although their association with Th phenotypes remained unclear in these studies. Nevertheless, this supports the immunodominant nature of these proteins and their role in inducing a Th-dependent antibody response, therefore rendering them excellent candidates for future experiments. Surprisingly, Hsp71-like protein (Ssa1) is among those proteins reactive only with Th2-dependent IgG1 antibodies, although Ssa1 has been reported to influence the immune response to *C. neoformans* during the afferent phase, but not during the efferent phase, eliciting no influence on adaptive immune response^33^. Additionally to antigens previously identified, this is the first time that Hsp72-like protein, a member of the highly immunogenic Hsp70 family^37^, thioredoxin-dependent peroxide reductase and two uncharacterized proteins (expressed protein Q5K7Y6, uncharacterized protein J9W025) are recognized as immunoreactive antigens in cryptococcal species. The identification of Th2-associated pathogenic proteins is of major therapeutic interest as a recent study could show that infection with a *C. neoformans* mutant strain lacking three chitin deacetylases and therefore chitosan, a component of the fungal cell wall and virulence factor, led to the development of a predominant Th1-type response and as a consequence to robust protective immunity if challenged with a *C. neoformans* wild-type strain^38^. Similarly, infection of mice with *C. neoformans* mutants characterized by a decreased Th2 bias after deletion of the respective genes, resulted not only in a prolonged survival of the animals but also in a predominant Th1-mediated immune response and decreased dissemination to the CNS, although in these cases prolonged immunity was not tested^30,31^. The proteins identified in our study may therefore serve as targets for the generation of *C. neoformans* knock-out mutants that could be used for similar vaccination-challenge experiments.

Three *C. neoformans*-specific antigens were found to be associated with a Th1 response as they reacted specifically with IgG2a antibodies (Table 2, Fig. 5). The protein phosphoglucomutase has been described so far only in *Aspergillus fumigatus* as an antigen expressed during invasive aspergillosis^39^. Vaccination with recombinant Hsp60-like protein has been associated with an improved course of disease in murine *Histoplasma capsulatum* infections^40^, underlining the potential protective influence of Hsp60-like protein also witnessed by its association with Th1-dependent IgG2a antibodies. Pyruvate decarboxylase is, to our knowledge, newly recognized as a fungal antigen. Further studies are necessary to test these IgG2a-reactive antigens in vaccination approaches for induction of a protective Th1 immune response. The identified IgG2a-reactive but *C. neoformans*-unspecific antigen ATP synthase subunit β could also be of particular interest for future studies, as this protein could be cross-reactive with different fungal species. Given the fact that this antigen was exclusively IgG2a-reactive and reactive with sera from all mice tested, indicating an immunodominant role, this protein could represent an excellent candidate for a protective vaccine against *C. neoformans* and potentially other fungal species. Previous studies demonstrated that immunization using protein fractions of *C. neoformans* and *C. gattii* prolongs the survival of mice against pulmonary cryptococcal infection^14,23^, but it has not been possible to elicit long lasting and effective protection. This suggests that a future vaccine should consist of fungal antigens selected for association with a protective Th1-response, rather than whole protein preparations.

Three proteins, Hsp90-like protein, spermidine synthase, and an uncharacterized protein (J9VHM5), were recognized by both IgG1 and IgG2a antibodies (Table 3, Fig. 5). Hsp90 has been identified as major immunogenic antigen not only in *C. neoformans*^13,14^ but also in *A. fumigatus*^39^. Reactivity with both isotypes could depend on a high fungal or microbial immunogenicity as evidenced by the high number of individual mice recognizing these antigens. Our study is the first to compare cryptococcal antigens recognized by sera of individual mice in contrast to other studies, which used pooled sera for their investigations. We found that several immunoreactive proteins, especially IgG1- or IgG1- and IgG2a-reactive proteins were observed with a high consistency throughout sera of individual mice. A likely explanation for this observation is the uniform major histocompatibility complex (MHC) haplotype (H-2^d^) of BALB/c inbred mice used in this study. Other immunoproteomic studies also used BALB/c mice^13,14^ for their experiments, which resulted in the identification of a number of identical antigens, underlining the importance of the MHC haplotype for antigen recognition by Th cells and development of antimicrobial antibodies. A recent immunization/challenge study proposed to combine multiple protein antigens in light of a critical role of MHC-II haplotype diversity for protection^41^.

We chose to identify immunoreactive cryptococcal antigens using sera from mice infected with *C. neoformans* for at least 56 days to mimic a prolonged interaction of the fungus with the immune system, as it occurs within the human population. We do not expect a different pattern of immunoreactive proteins in earlier stages of infection, as hallmarks of a Th2-polarisation, like IL-13 and IL-5 production as well as expression of GATA3 in Th cells are present in wild-type mice infected with *C. neoformans* already on day 21 post infection (dpi)^42^. Furthermore, there was no obvious influence of the susceptibility and fungal burden on the pattern of immunoreactive proteins identified, as we could observe several proteins recognized by sera from mice of all genotypes despite their underlying predominant immune response, different courses of disease and fungal burden in the lung.

Although most of the proteins identified in this study are associated with cytoplasmic functions, it is known that proteins like 14-3-3 protein, heat shock proteins, pyruvate decarboxylase, and phosphopyruvate hydratase (enolase) can be found in the cell wall of fungi^43,44^. The protein export mechanisms of these proteins may serve to promote microbial interaction with the host to stimulate an immune response. As previously reported in other studies, no mannoproteins were found to be immunoreactive with either IgG1 or IgG2a antibodies^14^, indicating that the method used for protein extraction in this study may underrepresent these scarce proteins or other immunoreactive proteins^45^.

To conclude, our study resulted in the identification of a significant number of antigens that are associated with Th2-dependent IgG1 antibodies and potentially may serve for fungus-specific immunotherapy strategies. In addition, selected antigens reactive with Th1-dependent IgG2a can be used for protective immunization experiments. Besides, some of the identified Th1- or Th2-associated serological antigen-specific responses may have the potential to be used as diagnostic markers to monitor the prognosis or antifungal treatment response of patients with cryptococcosis. At the same time, the finding of distinct IgG1- and IgG2a-immunoreactive fungal proteins provides molecular candidates to study immunomodulatory mechanisms of fungal antigens during the process of Th cell differentiation.

## Materials and Methods

### Sera collection

Serum samples, obtained after at least 56 dpi from wild-type and gene-deficient adult female BALB/c mice (H-2^d^) previously infected by nasal inhalation with a single inoculum of 500 colony forming units of *C. neoformans* strain 1841 (serotype D) yeasts^21^, were utilized through the study. Sera from infected immunocompetent wild-type mice, which have shown to develop a strong Th2 response with high levels of IgE^5,22^, were tested. In addition, sera from infected IL-12-deficient mice (IL-12p35^−/−^ and IL-12p40^−/−^), which present a strong Th2 biased immune response upon pulmonary infection with *C. neoformans*^21^ were included. Sera from infected IL-4Rα-deficient mice (IL-4Rα^−/−^), which show a reduced Th2-immune response in pulmonary cryptococcosis^20,46^ were also tested to enlighten the cryptococcal specific isotype production in a Th1 driven environment. All BALB/c wild-type mice succumbed to intranasal infection starting at 70 dpi (median survival time 74 dpi, unpublished data). In contrast, death of IL-12p35^−/−^ and IL-12p40^−/−^ mice started at significantly earlier time points (median survival time 52 dpi, unpublished data), whereas all IL-4Rα-deficient mice survived the pulmonary cryptococcal infection, but maintained detectable levels of cryptococcal cells in their lungs^20^. As negative controls sera from naïve mice of all three genotypes were used. Per group, 14 infected and 9–14 naïve mice from at least two different infection experiments were analysed. The mice were maintained under specific pathogen-free conditions, according to the guidelines authorized by the Animal Care and Usage Committee of the “Landesdirektion Sachsen” (www.lds.sachsen.de, Chemnitz, Germany) with food and water *ad libitum*. All infection experiments were carried out in accordance with the guidelines of the Committee of the “Landesdirektion Sachsen” according to the approved protocols with numbers 24-9168.11-TVV 5/01 and 24-9168.11 TVV 15/05.

### Protein extraction

*C. neoformans* strain 1841 was recovered from 10% fetal calf serum stocks stored at −80 °C and grown for 48 h in Sabouraud dextrose agar medium while shaking gently at 30 °C. For ELISA, yeast cells were harvested by centrifugation and washed twice with 250 mM sucrose. After washing, yeast pellets were resuspended in lysis buffer containing 10 mM Tris/HCl pH 7.5 supplemented with 5 mM EDTA and 1x protease-inhibitor cocktail (Roche, Basel, Switzerland). Thereafter, the suspension was transferred into a lysis-tube containing a mix of 0.1 mm glass beads together with 1.4 mm ceramic beads (PEQLAB, Erlangen, Germany) and cells were lysed by homogenization in the Peqlab-homogenizer at 4 °C (Precellys^®^ 24). The suspension was centrifuged twice transferring every time only the supernatant. The protein concentration was estimated using the Bradford reagent (Carl Roth, Karlsruhe, Germany) and samples were stored at −30 °C.

For one- and two-dimensional (1D and 2D) gel electrophoresis, some modifications were done to the protein extraction methodology in order to increase and maintain the solubility of the proteins. Yeast cells were harvested as previously mentioned and after washing, in addition to the lysis buffer, the pellets were mixed with an equal volume of a solution containing 8% CHAPS and 100 mM DTT. This suspension was disposed into a mortar and cells were frozen with liquid nitrogen and homogenized with a pestle twice. The homogenates were centrifuged and the protein suspensions were recovered. Protein content was estimated using the Bradford reagent (Carl Roth, Karlsruhe, Germany). Finally, proteins were precipitated overnight at −20 °C with 100% TCA (final concentration of 10% w/v) and washed three times with cold acetone to remove impurities or interfering substances. Pellet samples were kept at −30 °C until further analyses.

### Immunoglobulin isotyping

Total levels of Th2-dependent IgG1 and IgE and Th1-dependent IgG2a were determined in mice sera as previously described^5^. Briefly, 96 well round button plates were coated overnight at 4 °C with goat anti-mouse-IgG1, -IgE or -IgG2a, respectively (SouthernBiotech, Birmingham, AL, USA) in carbonate buffer. The plates were washed once with phosphate buffered saline (PBS) containing 0.05% Tween-20 (PBST) and blocked with PBS containing 0.5% BSA and 0.1% gelatine for 1 h at room temperature. Mouse IgG1, IgE, and IgG2a (SouthernBiotech, Birmingham, AL, USA), were used as standards, respectively. Sera were diluted in blocking buffer containing 0.05% Tween-20 up to 1:25,000 for IgG1 and IgG2a and up to 1:90 for IgE. The plates were incubated with the serum samples for 1.5 h at room temperature and washed three times with PBST. Detection was done with goat antibodies labelled with horseradish peroxidase (HRP) and specific for mouse IgG1, IgE, and IgG2a, respectively (SouthernBiotech, Birmingham, AL, USA), diluted 1:4,000. After 2 h incubation, the plates were washed four times with PBST and developed with 3,3′,5,5′-tetramethylbenzidine (KPL, Gaithersburg, MD, USA). Immediately after the wells with the higher concentration of the standard antibody reached an OD of 1.3 at 650/480 nm, developing of the plates was stopped by adding 1M H_3_PO_4_. A final reading of the plates was done at 450/630 nm and the concentration of each immunoglobulin isotype was calculated per serum sample^47^.

Additionally, titers of *C. neoformans-*specific IgG1 and IgG2a antibodies were determined for all serum samples as previously described^47^, with some modifications. ELISA plates were coated overnight with 0.5 µg of *C. neoformans* 1841 protein extract per well. Blocking was done with 5% skim milk dissolved in PBS (SM). Sera from infected mice were diluted in SM containing 0.05% Tween-20 (SMT) starting from 1:100 up to 1:409,600 for IgG1, due to expected higher titers, and starting from 1:50 up to 1:25,600 for IgG2a. Sera from naïve mice were diluted in SMT starting from 1:50 up to 1:25,600 for both isotypes. Detection was done with goat anti-mouse IgG1, human ads-HRP or goat anti-mouse IgG2a, human ads-HRP, respectively (SouthernBiotech, Birmingham, AL, USA). Development of the plates was done with 3,3′,5,5′-tetramethylbenzidine (KPL, Gaithersburg, MD, USA) for 45 min at room temperature and stopped with H_3_PO_4_ prior to OD determination at 450/630 nm^47^. The titer of *C. neoformans*-specific immunoglobulins was defined as the highest dilution at which the OD still showed a linear reduction. ELISA experiments to determine *C. neoformans*-specific IgE titers were not carried out, as previous studies of our group indicate that the expected concentration of *C. neoformans*-specific IgE is very low^22^. For all ELISA experiments, wells incubated without serum samples but with all other reagents were used as blanks. All experiments were done in technical duplicates.

### One-dimensional electrophoresis and immunoblot analysis

In order to assess the reactivity of serum IgG1 and IgG2a antibodies against specific cryptococcal proteins, one-dimensional (1D) SDS-PAGE and western blot were performed, according to methods previously described^48,49^. Briefly, *C. neoformans* protein pellets were dissolved in PBS to a final concentration of 1 mg/ml, mixed with the same volume of 2x Lämmli buffer and heated for 5 min at 95 °C. In each well of a 12.5% acrylamide gel 10 µg of protein were applied. Proteins were separated in Tris-glycine-SDS running buffer using the Owl™ Dual-Gel Vertical Electrophoresis Systems P8DS equipment (ThermoFisher Scientific, Waltham, MA, USA). For immunological detection, the separated proteins were transferred onto a nitrocellulose membrane by electroblotting using the Mini Trans-Blot equipment (BioRad, Hercules, CA, USA). After blotting, membranes were blocked overnight at 4 °C with 5% skim milk dissolved in distilled water (blocking buffer). Subsequently, membranes were incubated for 3 h at room temperature with sera from infected and naïve mice, respectively, diluted 1:1,000 in blocking buffer containing 0.1% Tween-20. Membranes were washed with PBST and incubated 1 h at room temperature with 1:4,000 goat anti-mouse IgG1 or goat anti-mouse IgG2a antibodies coupled to HRP (SouthernBiotech, Birmingham, AL, USA) to detect specific IgG1 or IgG2a antibodies, respectively, which bind to one or more *C. neoformans* proteins. Development of the membranes was done with SuperSignal® West Pico Chemiluminescent Substrate (ThermoFisher Scientific, Waltham, MA, USA).

### Two-dimensional electrophoresis

Sera with *C. neoformans* specific immunoglobulin levels near to the median values of all samples of a genotype, in order to guarantee representative results for all samples, were further analysed by 2D gel electrophoresis. Additionally, serum samples with high titers of specific antibodies for *C. neoformans* were also investigated by 2D gel electrophoresis to see if sera with high titers are reactive with an increased number of *C. neoformans* proteins. In total, five serum samples from infected wild-type mice and four sera from infected mice of each gene-deficient mouse strain were studied. From naïve mice, one serum sample per genotype was included.

Per gel, a pellet of 100 µg of *C. neoformans* proteins was resuspended in 125 µl of rehydration buffer (7M urea, 2M thiourea, 4% CHAPS, 50 mM DTT, 1% BioLyte^®^ (BioRad, Hercules, CA, USA), 0.001% bromophenol blue) and applied onto an IPG strip (BlueStrips 3–10 NL/7 cm, SERVA, Heidelberg, Germany). Strips were rehydrated for 6 h at room temperature and proteins were focused overnight using the PROTEAN IEF cell (BioRad, Hercules, CA, USA) under the following conditions: active rehydration, 50 V for 6 h; Step 1, 150 V, rapid ramp for 1 h; Step 2, 300 V, rapid ramp for 1 h; Step 3, 1,000 V, linear ramp for 1 h; Step 4, 3,000 V, linear ramp for 2 h; Step 5, 3,000 V, rapid ramp for 2 h; and Step 6, 500 V for 12 h. Following isoelectric focusing, strips were soaked twice in equilibration buffer containing 6M urea, 2% SDS, 50 mM Tris/HCl pH 8.8 and 20% glycerol for 15 min. For the first equilibration step 2% DTT was added to the equilibration buffer and for a second equilibration step 2.5% iodoacetamide was added to the equilibration buffer. After equilibration, strips were soaked briefly in Tris-glycine-SDS running buffer and placed separately on a 12.5% acrylamide SDS gel. Proteins were separated in a second dimension in the Owl™ Dual-Gel Vertical Electrophoresis Systems P8DS equipment (ThermoFisher Scientific, Waltham, MA, USA). Proteins in the gels were stained with Coomassie Brilliant Blue G250 dissolved in 10% acetic acid and 50% methanol and subsequently destained with a solution containing only 10% acetic acid and 50% methanol followed by washing with water, or alternatively transferred onto a nitrocellulose membrane for further detection of immunoreactive proteins, as described above. Membranes were incubated with sera from infected mice diluted 1:1,000 in blocking buffer containing 0.1% Tween-20. Sera from naïve mice diluted 1:500 were also tested.

### Identification of proteins by mass spectrometry

Spots of interest were mapped by overlaying Ponceau-stained nitrocellulose membranes, immunoblots and Coomassie-stained gels. Mapping was carried out with the software Delta2D (DECODON, Greifswald, Germany). The spots were excised manually from Coomassie-stained gels and digested *in situ* with trypsin. As described previously^50^, the resulting peptides were eluted out of the gel, concentrated by vacuum centrifugation, and analysed using a hybrid mass spectrometer (QExactive HF, ThermoFisher Scientific, Waltham, MA, USA) equipped with a chip-based electrospray device (TriVersa NanoMate, Advion) and coupled to a nano-ultra-performance liquid chromatography system (Dionex UltiMate 3000 RS, ThermoFisher Scientific, Waltham, MA, USA). A mass spectra (MS) database search was conducted using the MaxQuant software (version 1.4.1.2)^51^ against a concentrated UniProt database, which contains all reviewed and unreviewed *C. neoformans* proteins (crytococcusneoformans.uniprot.fasta). For the search, the following parameters were included: trypsin digestion, up to two missed cleavages, fixed modifications: carbamidomethylation as well as oxidation and the following variable modifications: first search peptide tolerance of 10 ppm, FTMS/MS/MS match tolerance of 10 ppm, a minimum of two peptides/protein, including at least one unique protein.

### Statistical analysis

Mann-Whitney U test was performed to determine the significance of the differences in the total level of immunoglobulins between wild-type, IL-12-deficient and IL-4Rα-deficient mice according with the ELISA results, as the data did not show a Gaussian distribution. Data are presented as individual points and medians. A nonparametric Spearman’s correlation test was done to determine the strength and direction of association between total and specific levels of IgG1 and IgG2a. The degree of significance was annotated as following: *p ≤ 0.05, **p ≤ 0.01, ***p ≤ 0.001, ****p ≤ 0.0001. GraphPad PRISM v7 software was used for statistical analyses (GraphPad Software, La Jolla, CA, USA).

### Data availability statement

The datasets generated during and/or analysed during the current study are available from the corresponding author on reasonable request.

## Electronic supplementary material

Supplementary Figures 1-4
