# Supplementary material for: Identification of T helper (Th)1- and Th2-associated antigens of Cryptococcus neoformans in a murine model of pulmonary infection
Source: Sci Rep. 2018 Feb 8;8:2681. doi: 10.1038/s41598-018-21039-z (PMC5805727; doi:10.1038/s41598-018-21039-z)
Supplement: Supplementary file 1 — Supplementary Figures 1-4 [file 41598_2018_21039_MOESM1_ESM.pdf]

Identification of T helper (Th)1- and Th2-associated antigens of *Cryptococcus neoformans* in a murine model of pulmonary infection

Carolina Firacative, A. Elisabeth Gressler, Kristin Schubert, Bianca Schulze, Uwe Müller, Frank Brombacher, Martin von Bergen, Gottfried Alber

Supplementary Information

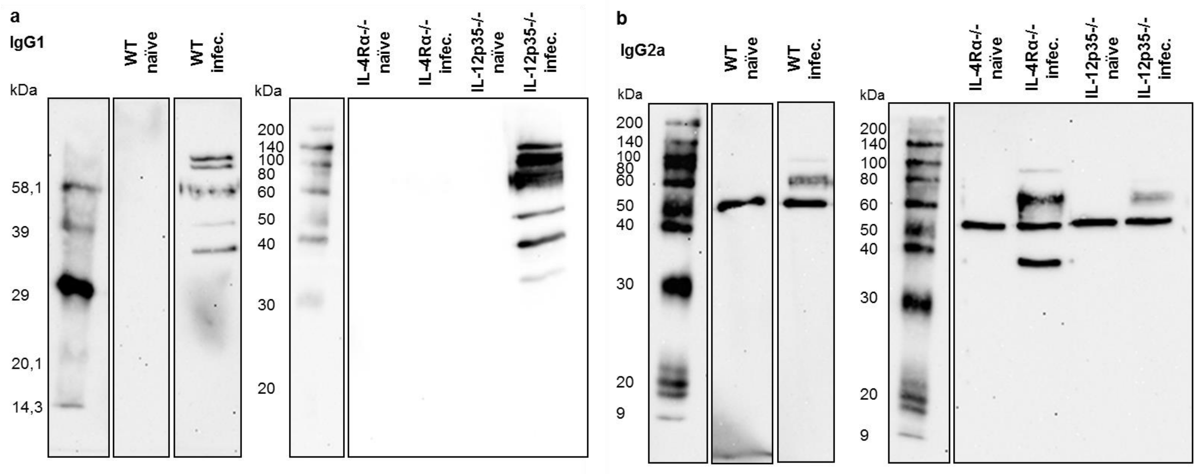

Supplementary Figure 1: Proteins of *Cryptococcus neoformans* strain 1841 separated by 1D gel electrophoresis react with antibodies from sera of infected and naïve wild-type and gene-deficient mice. Whole cell proteins of *C. neoformans* strain 1841 were separated by molecular weight for immunoblot analysis. After 1D gel electrophoresis, gels were transferred to nitrocellulose membranes. IgG1-immunoreactive proteins (A) and IgG2a-immunoreactive proteins (B) were detected using HRP-coupled secondary antibodies. Lanes of both membranes were incubated separately with sera from representative naïve and infected (infec.) wild-type (WT), an IL-12p35-deficient and an IL-4Rα-deficient (IL-4Rα$^{-/-}$) mice. Lanes were also incubated with sera from naïve mice of all genotypes. All sera were diluted 1:1,000. Images were cropped to improve clarity. Full-length blots are presented in Supplementary Figure 4.

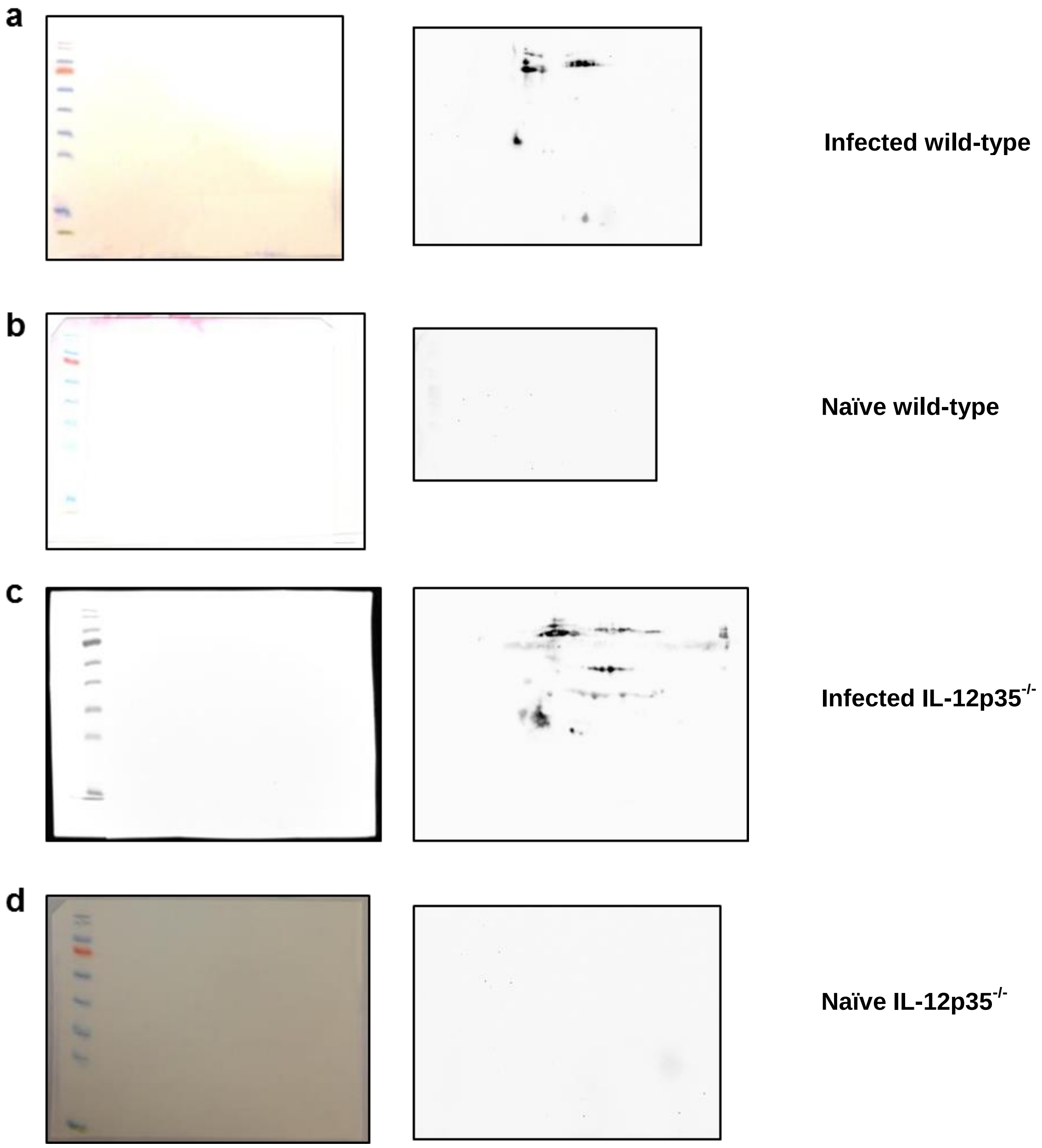

Supplementary Figure 2: Whole blots from Figure 2. IgG1-immunoreactive proteins from *Cryptococcus neoformans* detected with sera from representative infected but not naïve wild-type and IL-12-deficient mice. Whole cell proteins of *C. neoformans* strain 1841 separated by 2D electrophoresis were transferred to nitrocellulose membranes, which were thereafter incubated with sera from infected and naïve wild-type and gene-deficient mice diluted 1:1,000. IgG1-immunoreactive proteins were detected using sera from an infected wild-type (a), a naïve wild-type (b), an infected IL-12-deficient (c) and a naïve IL-12-deficient (d) mouse. Protein abundance seen in the Coomassie staining did not correlate with the strength of the immunoreactive signal (Fig. 4). Pictures of the whole membrane were taken with white light to visualize the pre-stained marker that was transferred to the membrane and recolored to grey scale when needed (left images). Immunoreactivity of the proteins with IgG1 was visualized with chemiluminescence (right images). The portion of the membrane with the marker was cropped, adjusted in size and overlapped with the whole membrane visualized with chemiluminescence.

a

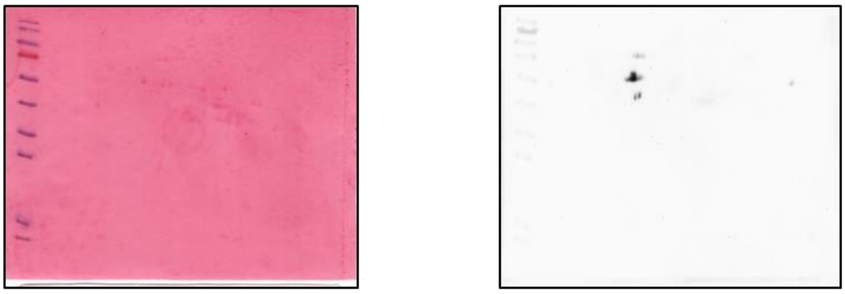

Infected wild-type

b

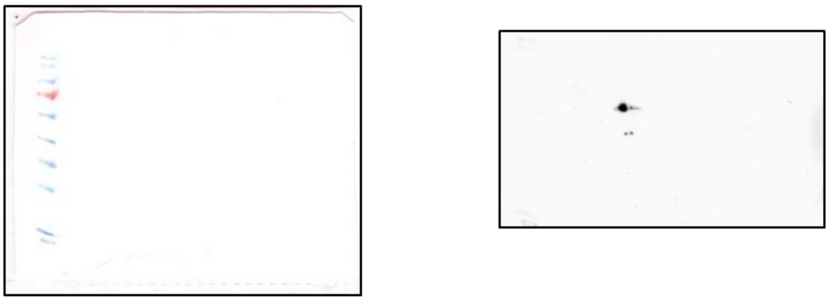

Naïve wild-type

c

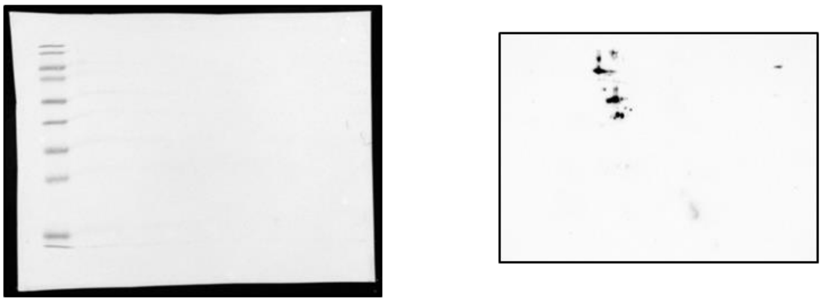

Infected IL-4Rα$^{-/-}$

d

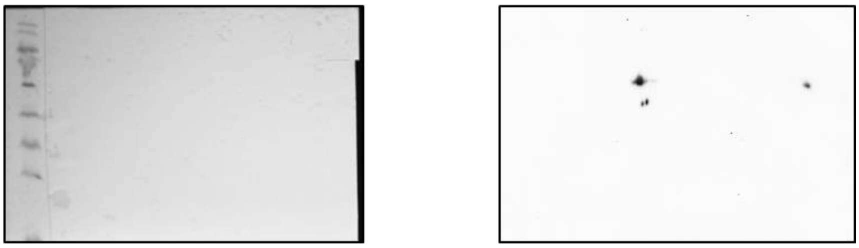

Naïve IL-4Rα$^{-/-}$

e

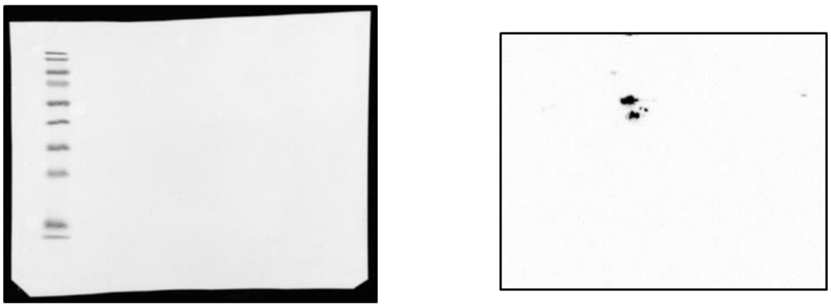

Infected IL-12p35$^{-/-}$

f

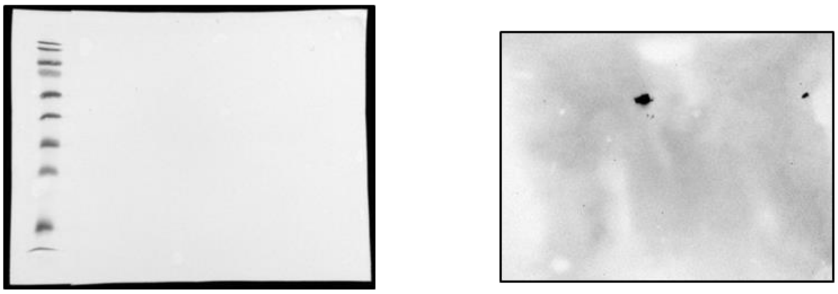

Naïve IL-12p35$^{-/-}$

Supplementary Figure 3: Whole blots from of Figure 3. IgG2a-immunoreactive proteins from *Cryptococcus neoformans* detected with sera from representative infected and naïve wild-type and IL-4Rα-deficient mice. Whole cell proteins of *C. neoformans* strain 1841 separated by 2D electrophoresis were transferred to nitrocellulose membranes, which were thereafter incubated with sera from infected and naïve wild-type and IL-4Rα-deficient mice diluted 1:1,000. IgG2a-immunoreactive proteins were detected using sera from an infected wild-type (a), a naïve wild-type (b), an infected IL-4Rα-deficient (c), a naïve IL-4Rα-deficient (d), an infected IL-12-deficient (e) and a naïve IL-12-deficient (f) mouse are shown. Protein abundance seen in the Coomassie staining did not correlate with the strength of the immunoreactive signal (Fig. 4). Pictures of the whole membrane were taken with white light to visualize the pre-stained marker that was transferred to the membrane and recolored to grey scale when needed (left images). Immunoreactivity of the proteins with IgG2a was visualized with chemiluminescence (right images). The portion of the membrane with the marker was cropped, adjusted in size and overlapped with the whole membrane visualized with chemiluminescence.

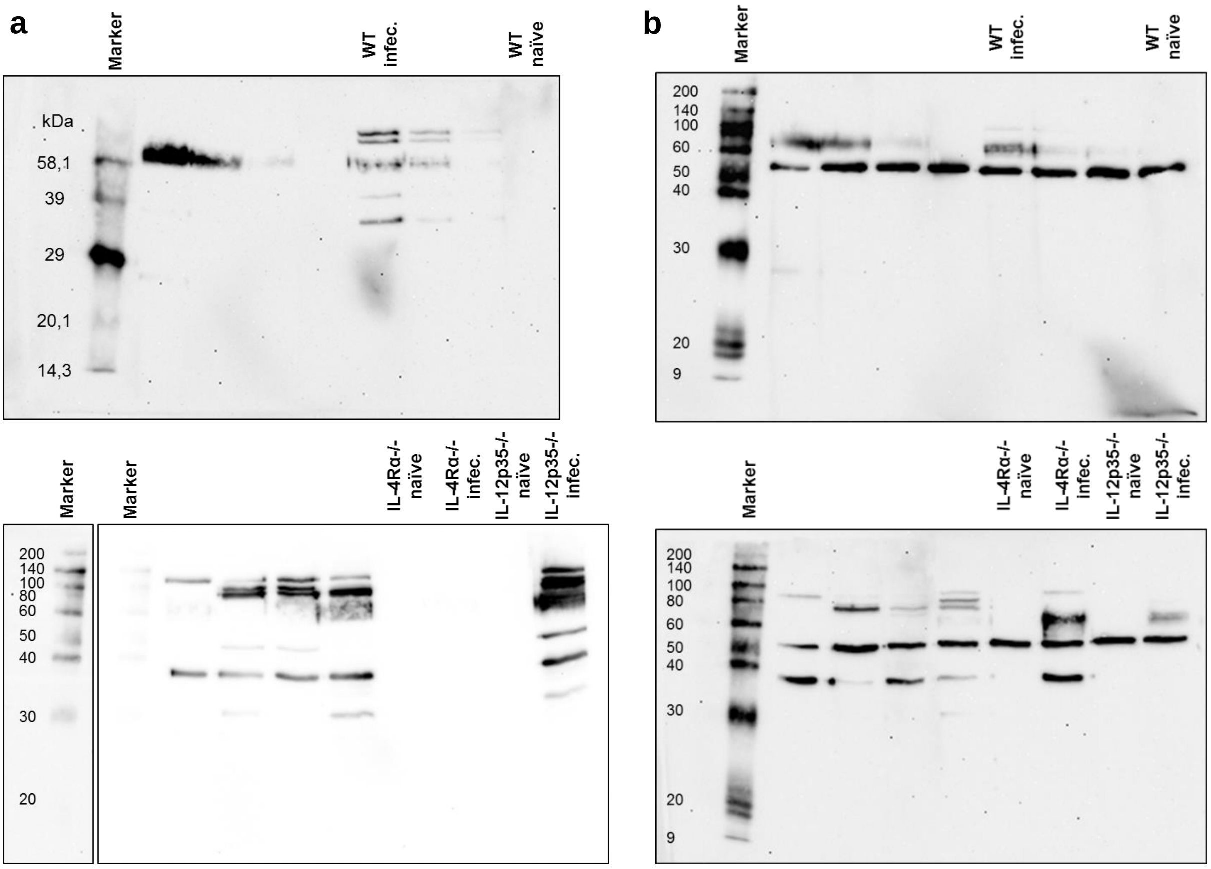

Supplementary Figure 4: Whole blots from Supplementary Figure 1. Proteins of *Cryptococcus neoformans* strain 1841 separated by 1D gel electrophoresis react with antibodies from sera of infected and naïve wild-type and gene-deficient mice. Whole cell proteins of *C. neoformans* strain 1841 were separated by molecular weight for immunoblot analysis. After 1D gel electrophoresis, gels were transferred to nitrocellulose membranes. IgG1-immunoreactive proteins (A) and IgG2a-immunoreactive proteins (B) were detected using HRP-coupled secondary antibodies. Lanes of both membranes were incubated separately with sera from representative naïve and infected (infec.) wild-type (WT), an IL-12p35-deficient (IL-12p35$^{-/-}$) and an IL-4Rα-deficient (IL-4Rα$^{-/-}$) mice. Lanes were also incubated with sera from naïve mice of all genotypes. All sera were diluted 1:1,000.
